# Chemometrics-Assisted Enhancement of Electrochemical Biosensor Performance toward miRNA Detection

**DOI:** 10.1021/acs.analchem.4c05402

**Published:** 2025-04-12

**Authors:** Wanda Cimmino, Simona Esposito, Panagiota M. Kalligosfyri, Nunzia Iaccarino, Stefano Cinti

**Affiliations:** † Department of Pharmacy, University of Naples “Federico II”, 80131 Naples, Italy; ‡ Sbarro Institute for Cancer Research and Molecular Medicine, Center for Biotechnology, College of Science and Technology, Temple University, Philadelphia, Pennsylvania 19122, United States

## Abstract

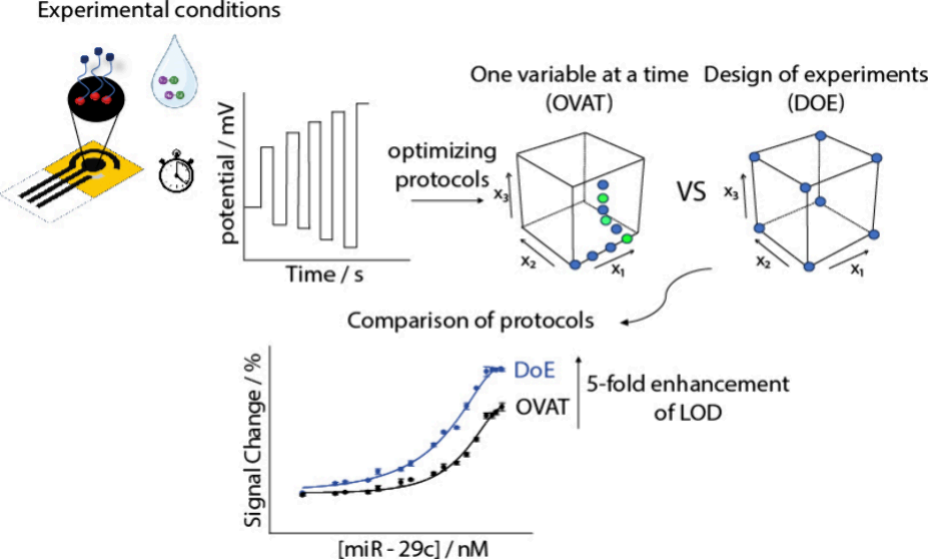

Chemometrics represents a potent tool for optimizing the experimental setup and subsequently boosting the performance of analytical methods. In particular, design of experiments (DoE) allows the experimental conditions to be optimized with high accuracy and a lower number of experiments when compared with the classical univariate approach, also known as one variable at a time (OVAT), which provides only a partial understanding on how factors affect the response. In this work, DoE was exploited, specifically a D-optimal design was used, to improve the analytical performance of a hybridization-based paper-based electrochemical biosensor, taking as target of the study the miRNA-29c (miR-29c) that is related to triple negative breast cancer. The sensing platform is composed of six variables to be optimized, including both those related to the sensor’s manufacture (i.e., gold nanoparticles, immobilized DNA probe) and those related to the working conditions (i.e., ionic strength, probe-target hybridization, electrochemical parameters). The adoption of DoE allowed us to optimize the device using only 30 experiments with respect to the 486 that would have been required with the OVAT approach, and as a consequence of the more accurate optimal conditions that have been reached, the detection of miRNA was more sensitive and repeatable when compared with previous data reported using the univariate approach for optimization, leading to a 5-fold limit of detection (LOD) improvement toward miRNA. It confirms that chemometrics might be considered a fundamental tool to be used in the development of various kinds of sensors and biosensors.

## Introduction

In recent years, liquid biopsy (LB) has emerged as a powerful tool in cancer diagnostics, enabling the noninvasive detection and monitoring of tumor biomarkers such as circulating tumor DNA (ctDNA),^1^ circulating tumor cells (CTCs), extracellular vesicles (EVs), and microRNAs (miRNAs).^2^ Monitoring these biomarkers may be crucial for early diagnosis and the follow-up of anticancer treatments.^3,4^ In particular, the possibility to detect these species in bodily fluids, including serum, whole blood, sweat, etc., makes sensors and biosensors promising analytical tools for use by nonspecialists and in decentralized settings. These tools are characterized by quickness, low-cost, and reliability, making them ideal for personalized treatment purposes.^5^

Among the existing portable analytical devices, electrochemical (bio)sensors have been highlighted due to their facile customization, wide application, high sensitivity and selectivity, and fast response time.^6^ However, the analytical performance of these platforms is strongly affected by the optimization of the various experimental conditions, which mainly include the modifiers, the physical parameters, the setup for the detection method, etc. Obtaining the optimal conditions for a certain analytical method is critical to its ultimate sensitivity and repeatability. According to the literature, two main approaches are reported to optimize the experimental parameters of an analytical method: (i) the one-variable-at-a-time (OVAT) approach and (ii) design of experiments (DoE).

The widely used method, which is related to the optimization of one parameter at a time while leaving the other parameters constant, is called the OVAT approach. Although the field of (bio)sensor development is largely characterized by this approach, some limitations should be highlighted: OVAT requires a high number of experiments (time-consuming), does not allow for the study of the (hidden) interactions among the variables, and risks missing the real optimum.^7,8^ For instance, some electrochemical sensors have been developed using this approach; however, since they were designed through an OVAT strategy, they often exhibit lower sensitivity and limited optimization of experimental conditions. For instance, a paper-based electrochemical biosensor for the detection of lung cancer-related microRNAs (miR-155 and miR-21) was developed using an OVAT approach.^9^ While this method allowed for the fabrication and preliminary optimization of the sensor, the obtained limits of detection (LODs)—12.0 nM for miR-155 and 25.7 nM for miR-21—remained relatively high. This highlights a key drawback of OVAT optimization, which considers only one variable at a time, neglecting possible interactions between factors and often leading to a suboptimal performance. Had the authors employed a DoE approach, they could have systematically explored the effects of multiple variables simultaneously, identifying true synergistic effects and uncovering the actual optimal conditions for each parameter. By considering variable interactions, DoE would have likely led to a more precise optimization, improving sensor sensitivity and achieving lower LODs.

On the other hand, chemometrics, which includes multivariate analysis and DoE, represents a potent alternative to be adopted in the field of portable diagnostics.^10^ In particular, DoE is mainly exploited to optimize experimental parameters of both unknown and known processes, synthesis, sensors, etc., reaching the “real” optimum based on the evaluation of all variables which are designed to be varying at the same time.^11^ There are various types of DoE, including screening designs like the Plackett–Burman design (PBD) or the Box–Behnken design (BBD), or DoE like the factorial designs and D-optimal (DO) designs that can be applied to different needs. The DO design, specifically, is advantageous because it focuses on maximizing the information obtained by minimizing the number of experimental trials. By selecting experimental conditions that maximize the determinant of the information matrix, it ensures more precise parameter estimation, making it particularly beneficial when optimizing a large number of variables.^12^ For these reasons, we selected the DO design in this study. This approach, widely used in experimental design, strategically reduces the number of experiments while preserving statistical efficiency, making it especially suitable for complex optimization processes. To better contextualize this choice, we next provide an overview of alternative experimental design strategies and their respective advantages and limitations. For instance, screening designs, such as PBD designs, use high (+) and low (−) levels for each factor, requiring only *N* + 1 experiments to evaluate *N* variables. This approach is efficient and allows users to quickly identify the most important parameters of a system with minimal experimentation. Furthermore, these designs are simple and cost-effective, as they allow for a minimal number of experiments. However, screening designs have significant limitations. While they can identify significant factors, they do not provide guidance on the optimal settings of these factors, making further experimentation necessary for their refinement. Moreover, they often do not consider interaction effects between variables and generally have a resolution lower than that of more complex experimental designs. Consequently, follow-up studies are essential to optimize and fully understand the relationships between the identified parameters.^13^ On the other hand, factorial designs are invaluable for evaluating the interactions between different variables and understanding how they influence each other.^14^ This ability is a significant advantage, as it provides insights that can lead to more effective optimization strategies. However, factorial designs can become complex and resource intensive when dealing with large numbers of variables, making them less practical for the optimization of too many parameters.

Therefore, using these designs for sensor optimization has considerable advantages. By use of chemometric techniques, the optimization process can be simplified, leading to faster development cycles and improved sensor performance. For example, RSM based on central composite design (CCD) was used for the optimization of an electrochemical sensor for heavy metal detection.^15^ In this work, the researchers highlighted how, using DoE, they were able to optimize the performance of the sensor with only 13 experiments and improve its performance by achieving a lower detection limit than those previously reported in the literature (from 12 to 1 nM). In another work, an immunosensor for human epididymis protein 4 (HE4) detection in serum was optimized via a two-factor and three-level experimental design. The optimized parameters included the concentration of HE4 for magnetic bead functionalization and the concentration of anti-HE4 for the competition assay.^8^ Also, in this case, thanks to the experimental design, the researchers achieved a detection limit in the picomolar level, suitable for diagnostic application. Another example on the use of the experimental design for the optimization of an electrochemical strip is shown in the work of ref (16). In this case the researchers utilized a DO design to optimize two variables on three levels and two on four levels using only 19 experiments compared to the 108 required with the OVAT approach. In another work a full factorial design was utilized for the optimization of an electrochemical glucose biosensor: by using only 17 experiments, a sensitive and affordable device was optimized, also by decreasing the amount of dried reagents and by increasing the sensor’s stability based on previous studies.^17^ Thanks to the experimental design, the group was able to achieve similar results in current density by using 93% less nanoconjugate to manufacture the sensor and to improve the operational stability of the sensor by going from 50% to 75% amperometric current retained after 12 h of use.

**Figure 1:**
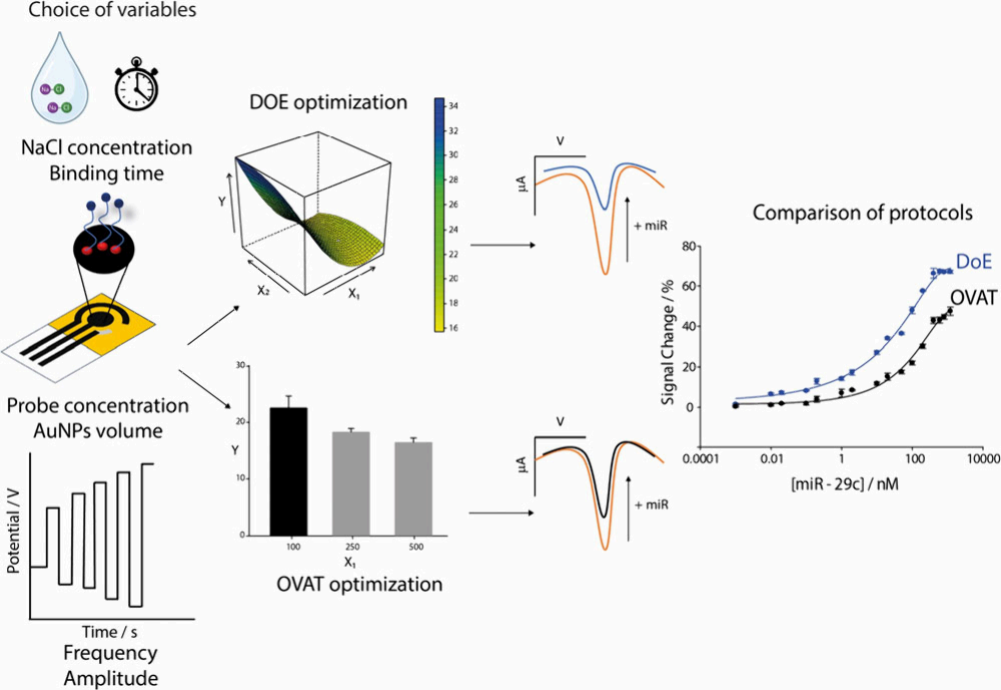
Schematic representation of the comparison between an optimization following DoE and the same platform optimized following OVAT.

These examples show that multivariate approaches offer significant advantages in the manufacturing and application of electrochemical (bio)sensors. However, when many parameters need optimization, these methods become less suitable due to the high number of required experiments. For instance, a typical full factorial design could be adopted for optimization. It works using 2^*k*^ experiments, where 2 is the level for all variables and *k* is the number of variables: if three variables need to be optimized, eight experiments will be carried out. Alternatively, if six variables need to be optimized, 64 experiments will be required.^18^ When each variable has more than two levels, the number of required experiments increases, making the optimization process longer and potentially less accurate. Among the various DoE approaches, DO designs can be particularly advantageous when optimizing multiple variables with several levels, as they allow for a high number of experiments while maintaining efficiency. DO is a tailored design that balances information gain and experimental effort by maximizing the normalized determinant of the information matrix, ensuring optimal analytical performance. As shown in the reported work of the Citterio group,^19^ a DO design enabled the optimization of a colorimetric biosensor through the identification of seven variables with only 44 experiments, and this approach resulted in being more efficient concerning other DoE such as the CCD and the Box–Behnken, characterized by 80 and 58 experiments, respectively. Therefore, due to the extensive scientific interest focused on the development of electrochemical biosensors for the application in the field of liquid biopsy,^6,20−24^ the aim of this study was to demonstrate for the first time how the adoption of DoE, in particular a DO design, can effectively improve the analytical performances of an electrochemical biosensor toward the detection of miRNA associated with cancer diagnosis/prognosis. A paper-based electrochemical biosensor, where the analytical response is due to the hybridization between a DNA-probe and miR-29c, was taken as the case of study. Six variables, including both manufacturing and chemical-physical ones, were considered to improve the analytical performance in comparison with the same biosensor optimized by following the OVAT approach (Figure 1). The choice of DO as the DoE allowed us to reach the optimal experimental conditions by performing only 30 experiments against the hypothetical 486 experiments required through the whole OVAT strategy. As the output, the adoption of DO was consistent with a 5-fold improvement of the analytical performance of the paper-based biosensor for miRNA detection with respect to the non-DoE optimized one. It represents an additional possibility to be applied within the field of biosensors to make existing approaches more effective, particularly in the case of liquid biopsy, where sensitivity is a crucial feature.

## Experimental Section

### Reagents and Instruments

All of this information is reported in the Supporting Information (SI).

### Preparation of the Electrochemical Strip and Measurement Principle

The electrochemical strip for miR-29c detection was developed as reported previously from the authors.^25−27^ All of the information is reported in the SI.

### Choice of Experimental Variables to Optimize

To optimize the electrochemical platform for miRNA detection, six key variables influencing the biosensor performance were considered. These were selected based on their relevance to fabrication, measurement, and electrochemical parameters in square wave voltammetry (SWV) (Table S1). Starting from the fabrication of the biosensor, the concentration of the capture probe was selected for optimization studies. A wide range of probe concentrations, particularly 100, 500, and 1000 nM, was considered to find the best compromise in terms of obtained signal and target affinity. Another important parameter evaluated was the amount of gold nanoparticles (AuNPs) used to modify the working electrode area. It was chosen to test 2, 8, and 15 μL of AuNPs to determine how they affect both the immobilization efficiency and the resulting electrochemical signal. Regarding the measurement step, the chosen parameters were the concentration of sodium chloride in the working solution, testing 50, 500, and 1000 mM, and the hybridization time, testing 10, 30, and 60 min. Finally, the variables regarding the electrochemical technique were chosen for optimization studies: the amplitude, tested on two levels 0.01 and 0.04 V, and the frequency, tested on three levels 10, 50, and 100 Hz, of the SWV. Amplitude refers to the height of the square wave potential signal applied to the working electrode. The amplitude influences the shape of the peak current obtained; in fact, with a high value of amplitude, it is possible to increase the peak current obtained, but too high of amplitudes may lead to peak broadening and decreased resolution. Frequency, on the other hand, determines the rate at which the potential is applied to the electrode, so higher frequencies decrease the measurement time and can give higher peaks, but very high frequencies can affect the resolution of the peaks, obtaining a higher noise.^28,29^

### Experimental Design

The optimization of the experimental parameters was attempted by using a D-optimal design, described by the following mathematical model:
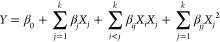
where *Y* is the predicted response of the biosensor in terms of signal change % between the measurement in the absence and presence of miR-29c; β_0_, β_*j*_, β_*ij*_, and β_*jj*_ are the regression coefficients for the intercept, linearity, interaction terms, and square, respectively; and *X*_*i*_ and *X*_*j*_ are the coded independent variables. To evaluate all 17 coefficients, it was decided to run 30 experiments. Indeed, as shown in the plot reported in Figure S1, the point that indicates 30 experiments represents the best compromise between the logarithm of the normalized determinant, which reflects the information retrieved, and number of experiments to be performed (experimental effort).

## Results and Discussion

### Evaluation of Model Coefficients and Response Surface

**Figure 2:**
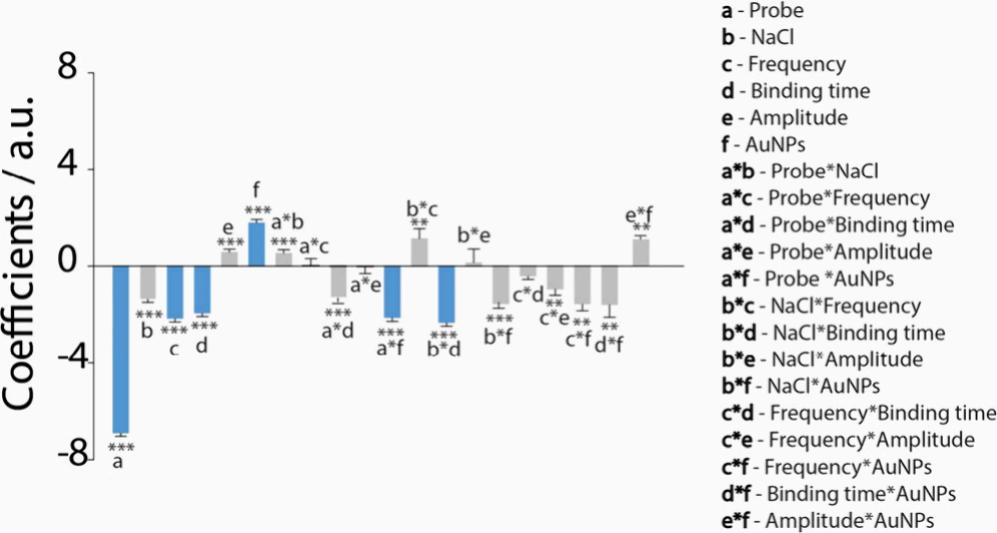
Plot of the model coefficients including the linear terms (a–f) and interaction terms. Highlighted in blue are the parameters with the greatest impact for the development of the platform. The level of significance is shown by using the star code (*p < 0.05, **p < 0.01, ***p < 0.001). Negative coefficients indicate inverse relationships, while positive coefficients suggest direct relationships with the response variable.

The optimization process is characterized by three sections: (1) performing statistical tests, (2) estimating coefficients in a mathematical model, and (3) projecting the response and verifying the adequacy of the model. So, once the variables and levels to be optimized were chosen, the experiments to be carried out were chosen by DO design, which were 30 against 486 candidate points. Once all the experiments were performed, the coefficients β of the model were evaluated. The magnitude of each β coefficient illustrates the impact or significance of each factor and interaction. As can be seen from Figure 2, the most important contributions are due to those variables and interactions that are characterized by high values of the histogram.

As shown in Figure 2, the probe concentration, the frequency applied during the measurements, the binding time, the amount of AuNPs, and the interactions between probe concentration and AuNP amount and between NaCl concentration and binding time emerged as the most critical factors for further optimization. Among these, probe concentration proved to be particularly significant, as it directly influences biosensor performance. From the DoE analysis, it emerged as a key variable due to its impact on both signal intensity and target affinity. The literature suggests that while increasing the DNA probe concentration can enhance the current signal, it may also reduce target affinity.^30^ Regarding the AuNPs, the coefficient analysis confirmed their significant role in biosensor optimization. As shown in Figure 2, a strong interaction was observed between AuNP amount and probe concentration. This can be explained by the function of AuNPs in facilitating probe immobilization on the electrode surface. By increasing the surface density of the immobilized DNA probe, AuNPs enhance the hybridization efficiency and amplify the electrochemical response, improving the signal obtained from methylene blue (MB) reduction.^31−33^ Another interaction to consider is between the NaCl concentration and binding time, both essential for target-probe hybridization. The NaCl concentration in the solution is important because it plays a crucial role in the hybridization processes. Higher ionic strength stabilizes interactions between negatively charged DNA strands, enhancing hybridization specificity while reducing nonspecific binding.^34^ In the same way, the hybridization time influences how effectively the target binds to the DNA probe, impacting overall the sensitivity of the platform. Contrarily, the amplitude of the SWV technique is characterized by a low histogram, indicating a low effect on the response of the biosensor and subsequently not interesting to be taken into account for further optimization.

**Figure 3:**
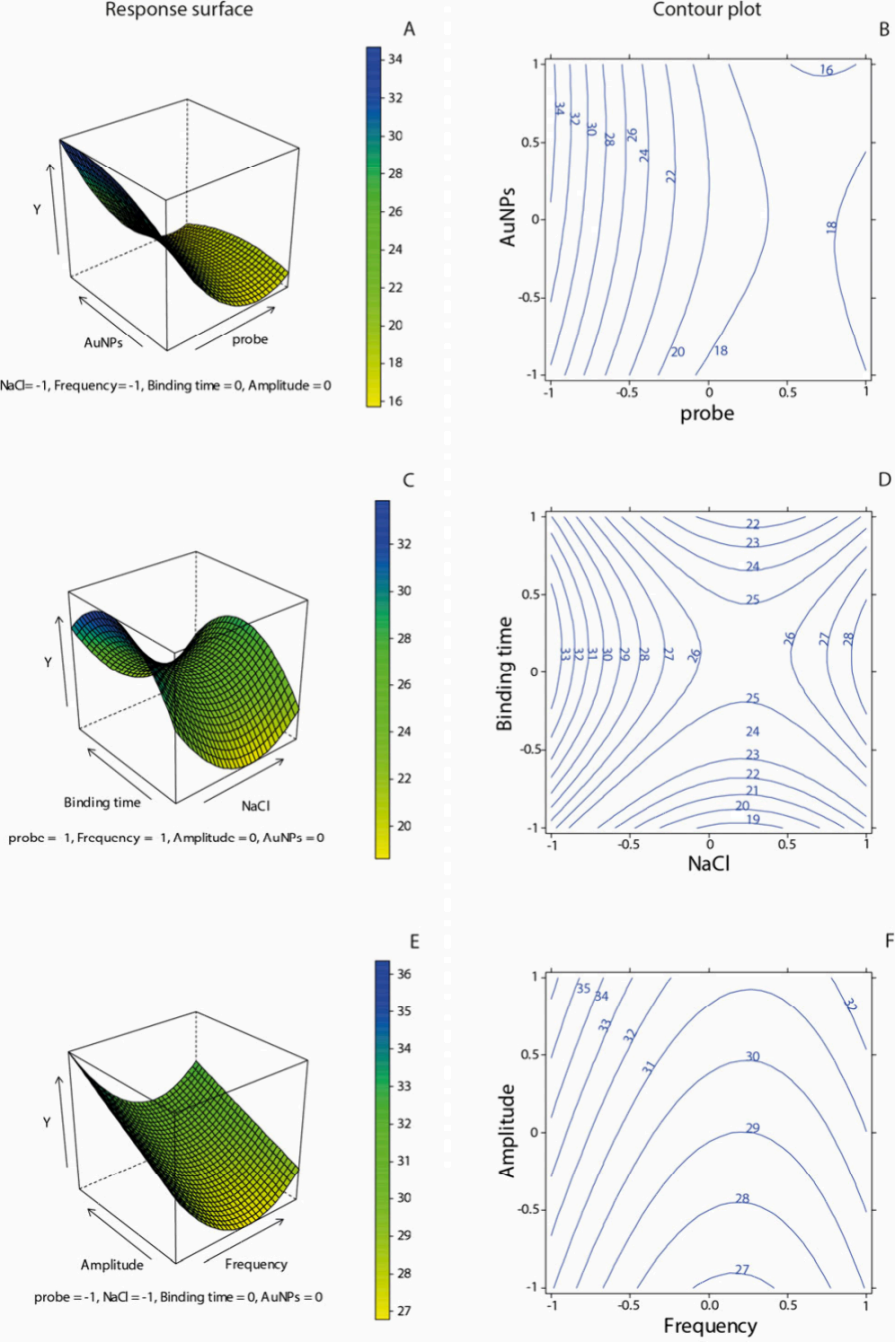
Response surface 3D and contour plots 2D of the signal change (%) as a function of the following variables: (A) 3D response surface plot of probe concentration and amount of AuNPs. (B) 2D contour plot of probe concentration and amount of AuNPs. (C) 3D response surface plot of binding time and NaCl concentration. (D) 2D contour plot of binding time and NaCl concentration. (E) 3D response surface plot of SWV frequency and amplitude. (F) 2D contour plot of SWV frequency and amplitude. In all of the response surface plots, the lower response is shown in yellow, while the higher response is represented in blue. In the contour plots, the values of −1, 0, and 1 indicate the coded levels of all variables. All plots depict the predicted signal change (%) in the presence of 50 nM target solution across the entire experimental domain.

Subsequently, the response surfaces were evaluated to choose the best value for each parameter, as shown in Figure 3, where response surfaces and contour plots are reported. Response surfaces are graphical representations of how a dependent variable changes with respect to two or more independent variables. They provide a visual way to explore the relationships between these variables and the response, allowing for the identification of optimal conditions. To create a response surface plot, data from experiments are fitted to a mathematical model. This model describes how the response variable is affected by the independent variables. The resulting surface shows the predicted values of the response variable with different combinations of the independent variables. Contour plots complement response surfaces by providing a two-dimensional view of the same data. They show lines connecting points with equal response values, making it easier to visualize regions of interest. Each contour line represents a specific value of the response variable, allowing for quick identification of optimal conditions.^35^

When evaluating these plots to select the best variable values, one looks for the optimal peaks or regions on the response surface where the desired outcome is maximized (or minimized). By analyzing both the response surface and the contour plots, it is possible to determine which combination of independent variables gives the best results for the response variable. This process is crucial in experimental design, as it helps to optimize conditions efficiently and effectively.

Figure 3A,B illustrates the effect of probe concentration and AuNP amount on the signal change (%). As the probe concentration decreases, probe-target affinity improves. Conversely, increasing the amount of AuNPs enhances the platform response. Figure 3B shows a greater signal change at low probe concentrations, particularly at the coded level of −1 (100 nM), where the response ranges between 30% and 34%. Additionally, a higher AuNP volume leads to an improved signal. Based on these results, 11.5 μL of AuNPs and a 100 nM probe concentration were selected for electrode modification. The amount of AuNPs plays a crucial role in both the electron transfer at the electrode surface and the immobilization efficiency of the DNA probe, ultimately influencing the sensor’s response signal. AuNPs enhance electron transfer by providing a highly conductive surface that facilitates the charge transport between the electrode and electroactive molecules. However, excessive AuNP deposition can lead to aggregation or multilayer formation, which may block direct electron communication with the electrode and increase resistance, reducing the efficiency of charge transfer. In terms of probe immobilization, a higher density of AuNPs provides more binding sites for thiol-modified DNA probes, improving probe immobilization and target hybridization efficiency.^36^ Therefore, the optimization of AuNP volume is essential to achieving a balance between maximizing probe immobilization, maintaining efficient electron transfer, and ensuring a strong and stable electrochemical signal. The selected volume of 11.5 μL significantly enhances sensor performance by increasing the electrode surface coverage, providing more immobilization sites for the probe, and expanding the electroactive area. This interaction between the probe density and the amount of AuNPs is crucial for improving the sensitivity of the platform and ensuring efficient electrochemical reduction of MB.

Figure 3C,D illustrates the effect of binding time and sodium chloride concentration in the working solution on the sensor’s response. The results indicate that the optimal hybridization time is around 30 min. Additionally, the response increases at moderate NaCl concentrations, suggesting that hybridization benefits from optimized ionic strength. Too low of NaCl concentrations may lead to inadequate charge screening, hindering probe-target binding, while excessively high NaCl concentrations may cause strong ionic interactions that can interfere with the hybridization efficiency. Binding time also plays a crucial role in the response: longer incubation periods allow for more stable hybridization, enhancing target recognition and signal output. However, excessive binding times may promote nonspecific interactions, reducing sensor specificity. The time required for probe-target complex formation is influenced by the sequence characteristics, including length and composition, as well as the equilibrium between the free and hybridized states. Literature reports suggest that hybridization of sequences around 20 base pairs typically occurs within 15–20 min.^37^ Based on these findings, the optimal parameters selected for the measurement step are 50 mM sodium chloride in the working solution and a binding time of 30 min, ensuring efficient and specific hybridization.

Regarding the SWV parameters, the response surface and the contour plot of the interaction between the frequency and amplitude to be applied during the measurements were evaluated. It can be noticed from Figure 3E,F that the best results in terms of signal change % were obtained with high values of amplitude, between 0.03 and 0.04 V, and low frequencies. The optimized parameters in this case were 0.036 V for the amplitude and 10 Hz for the frequency.

### Model Validation

For the validation of the experimental model, the results obtained experimentally were compared with those predicted at four points in the experimental domain. The variable’s levels utilized for each experiment are shown in Table S3. The predicted values were calculated based on the mathematical model describing the experimental domain, given in the SI. To obtain the predicted value of *Y* at a specific point, the known coefficients and corresponding values of the variables were substituted into the equation. By solving this equation with the appropriate values, the predicted response was derived for each combination of factors explored in the model validation experiments. In all cases, the experiments were carried out in triplicate, and the residuals and accuracy of the prediction were calculated, which turned out to be −0.13 and 99%, for point A, 0.54 and 98% for point B, - 0.88 and 98% for point C, and 0.14 and 99% for the last point D. As shown in the histograms in Figure S2, in all cases, the experimental results match the predicted results, and thus, the model is validated.

### Analytical Performances of the Platform

**Figure 4:**
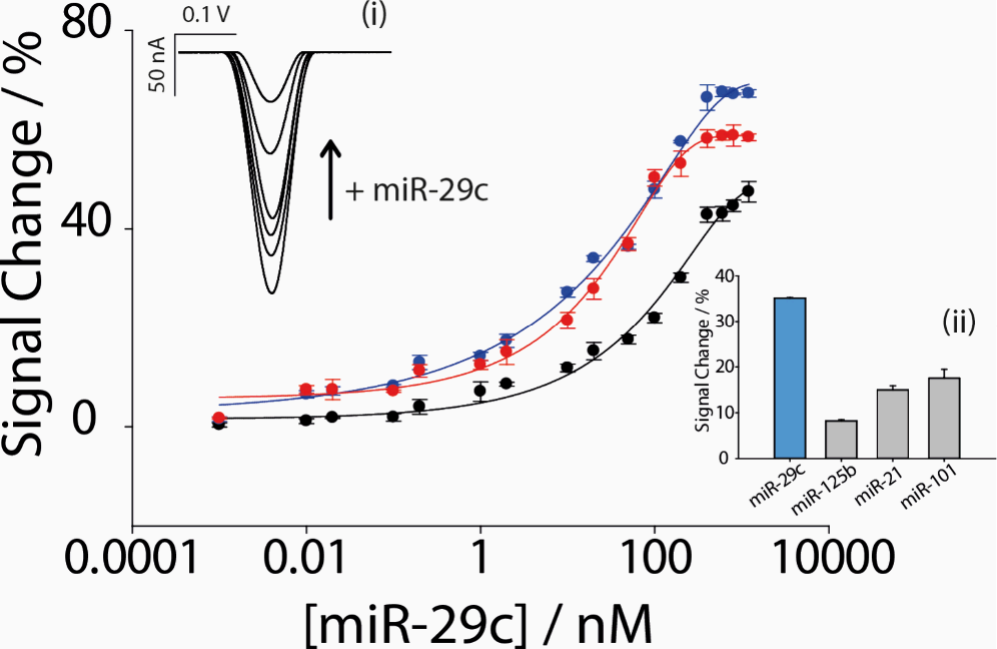
Signal change (%) of different increasing concentrations of miR-29c in nM range on a logarithmic scale. In black, signal change (%) obtained in PBS with the platform optimized with OVAT; in blue, signal change (%) obtained in PBS with the platform optimized in DOE; and in red, signal change (%) obtained with the platform optimized with DOE in commercial human serum. Insets: (i) SWV image of different increasing concentration of miR-29c; (ii) interference study of miR-29c in the presence of other miRNAs (miR-125b, miR-21, miR-101). All the experiments were carried out in triplicate.

The analytical performances of the electrochemical biosensor optimized in this work were tested in phosphate buffer and commercial human serum. In addition, to demonstrate the effectiveness of the experimental design, the analytical performances of the optimized platform were compared with those of the same platform optimized in our previous work^25,27,30^ with an OVAT approach. The OVAT-optimized parameters were as follows: probe 100 nM, NaCl 140 mM, frequency 50 Hz, binding time 30 min, amplitude 0.01 V, and AuNP volume 8 μL. The target concentration tested was in the range between 0.001 and 1200 nM. As can be seen in Figure 4, a semilogarithmic sigmoidal correlation emerged between the logarithmic scale of target concentration and the signal change %.

The limit of detection, defined at 10% of the maximum signal variation, was ∼1 nM for the OVAT-optimized platform and 0.2 and 0.3 nM for the DoE-optimized biosensor in phosphate buffer and human serum, respectively. The curve shows very similar performances in terms of repeatability, showing an RSD% lower than 5%. The selectivity study was performed in the presence of three random noncomplementary miR sequences: miR-125B-5p (5′-ucc cug aga ccc uaa cuu gug a-3′), miR-101-5p (5′-cag uua uca cag ugc uga ugc u-3′), and miR-21-5p (5′-uag cuu auc aga cug aug uug a-3′), tested at the concentration of 50 nM in phosphate buffer. As can be seen from the results in the inset of Figure 4, the platform shows a good selectivity for the target miR-29c, showing the ability of the platform to identify the target of interest.

## Conclusion

In this technical note, the analytical performance of an electrochemical biosensor for the detection of miRNA was successfully maximized by adopting a D-optimal design as the DoE for optimizing the factors involved in the manufacturing of the strip and analysis of the target. With the adoption of chemometrics, the biosensor was improved ca. 5-fold in terms of LOD with respect to the same device that was optimized following the univariate OVAT approach. In particular, DoE allowed us to obtain the “real” optimal experimental conditions that cannot always be obtained by optimizing each parameter and fixing the others. The chemometrics model was validated and successfully carried out to detect miR-29c down to ca. 0.2 nM in both standard and human serum solutions, with respect to the ca. 1 nM detection limit that was obtained by following the OVAT optimization approach of the variables. Even if chemometrics adoption in the field of biosensors is not prevalent yet, it represents a powerful tool to be applied for both optimizing platforms and analyzing complex results and hidden interactions, with the potential to improve the existing platforms in all fields of interest.

## Supporting Information Available

Information regarding the reagents and instruments that have been utilized, the preparation of the electrochemical strips, and all information regarding the experimental design: choice of variables and levels, the mathematical model, all the experiments carried out, and information regarding the model validation (PDF)
