# Supplementary material for: Chemometrics-Assisted Enhancement of Electrochemical Biosensor Performance toward miRNA Detection
Source: Anal Chem. 2025 Apr 12;97(15):8182–8. doi: 10.1021/acs.analchem.4c05402 (PMC12019774; doi:10.1021/acs.analchem.4c05402)
Supplement: Supplementary file 1 — ac4c05402_si_001.pdf [file ac4c05402_si_001.pdf]

Chemometrics-assisted enhancement of electrochemical biosensor performance towards miRNA detection

Wanda Cimmino[1], Simona Esposito[1], Panagiota M. Kalligosfyri[1], Nunzia Iaccarino[1], Stefano Cinti[1,2*]

1Department of Pharmacy, University of Naples "Federico II", 80131 Naples, Italy

2Sbarro Institute for Cancer Research and Molecular Medicine, Center for Biotechnology, College of Science and Technology, Temple University, Philadelphia, PA 19122, USA

* Corresponding author: Stefano Cinti, stefano.cinti@unina.it

Contents

Abstract S1
Reagents and equipment S1
Preparation of the electrochemical strip and measurement principle S1

Choice of experimental variables to optimize S2

Experimental design S2
Model validation S4

Abstract

Here are reported the information about the reagents and instruments used, the preparation of the electrochemical strips, all the information about the experimental design; the choice of variables and levels, the mathematical model, all the experiments carried out and the information regarding the model validation.

Reagents and equipment

Phosphate buffer, sodium chloride (NaCl), 6-mercapto-1-hexanol (MCH, $C_6H_{14}OS$), tris(2-carboxyethyl) phosphine (TCEP; $C_9H_{15}O_6P$), sodium borohydride, chloroauric acid ($HAuCl_4$), sodium citrate, and human serum were purchased from Sigma-Aldrich (St. Louis, MO, USA). The specific probe (5'-Thiol-C6- gaa cac cag gag aaa tcg gtc a- Atto MB2 - 3'), the target miR-29c – 5p (5' - uga ccg auu ucu ccu ggu guu c – 3') and the miR sequences utilized for the selectivity study: miR-101-5p (5'- cag uua uca cag ugc uga ugc u - 3'), miR-21-5p (5′- uag cuu auc aga cug aug uug a - 3') and miR-125B-5p (5'-ucc cug aga ccc uaa cuu gug a - 3') were purchased from Metabion GmbH (Steinkirchen, Germany). The office paper-based screen-printed electrodes was home produced by a semi-automatic screen printer, with two types of conductive inks: a silver ink (Loctite Italy) used for the production of the reference electrode and graphite ink (Sunchemical, USA) used for the production of the counter and the working electrode. All the measurements were carried out with a portable potentiostat Palmsens 4 (Palmsens, Netherlands) equipped with a multi-8 channel reader interfaced with a laptop using PSTrace 5.10 software. The data analysis was carried out utilizing the Chemometrics Agile Tool (CAT), an open-source software that can be found in the following link http://gruppochemiometria.it/index.php/software.

Preparation of the electrochemical strip and measurement principle

The immobilization of the capture probe on the working electrode surface area was carried out following four main steps. 1) The working electrode surface area was modified, by drop casting, with different amounts of AuNPs to allow the binding between the thiol, at the 5' end of the ss-DNA sequences, and the gold surface. 2) The capture probe was reduced in the presence of 0.01 M of TCEP for 1 h at room temperature in the dark. 3) A drop of 20 µL of different concentration of reduced probe was dropped on the working electrode for 1h in a humidity chamber. 4) The working electrode was incubated for 1.5 h in the presence of MCH in a humidity chamber to passivate the electrode surface and to define the orientation of the immobilized probe. MCH plays a crucial role in hybridization assays by serving as a passivating and blocking agent on the electrode surface. It helps orient DNA probes effectively while preventing nonspecific adsorption of interfering molecules such as proteins or small molecules. By forming a self-assembled monolayer, MCH minimizes direct access of these interferents to the electrode, thereby enhancing assay specificity and stability. Additionally, MCH ensures optimal probe immobilization, creating a well-structured surface that facilitates efficient DNA hybridization. The electrochemical measurement of miRNA in the solution is based on the signal-off architecture [7]. The presence of miRNA-29c, which has hybridized with the corresponding DNA probe immobilized on the working electrode, is associated with the change of the signal. In fact, when the target is present in the analyzed solution it binds to the probe and we record a lower current signal, than in the measurement made in the absence of the target. In order to evaluate this signal change in

the presence of the target, the electrochemical measurements were performed using square wave voltammetry. Briefly, 100 μL of drop was placed on the sensor after a time for stabilization of the probe the electrochemical measurement was performed, after which different concentrations of miRNA were added to the solution and after a time for hybridization another measurement was performed. The results are evaluated in terms of signal change % following the equation:

$$Signal\ change\% = \frac{I_{blank} - I_{target}}{I_{blank}} * 100$$

where I_{blank} is the current recorded in the absence of the target and I_{target} is the current recorded in the presence of the miR-29c.

Choice of experimental variables to optimize

Table S1: Experimental variables and levels. Five variables were evaluated on three levels and one variable was evaluated on two levels.

		Levels		
Independent variable	Symbol	-1	0	1
[Probe]	X_1	100 nM	500 nM	1000 nM
[NaCl]	X_2	50 mM	500 mM	1000 mM
Frequency	X_3	10 Hz	50 Hz	100 Hz
Hybridization time	X_4	10 min	30 min	60 min
Amplitude	X_5	0.01 V		0.04V
AuNPs	X_6	2 μL	8 μL	15 μL

Experimental design

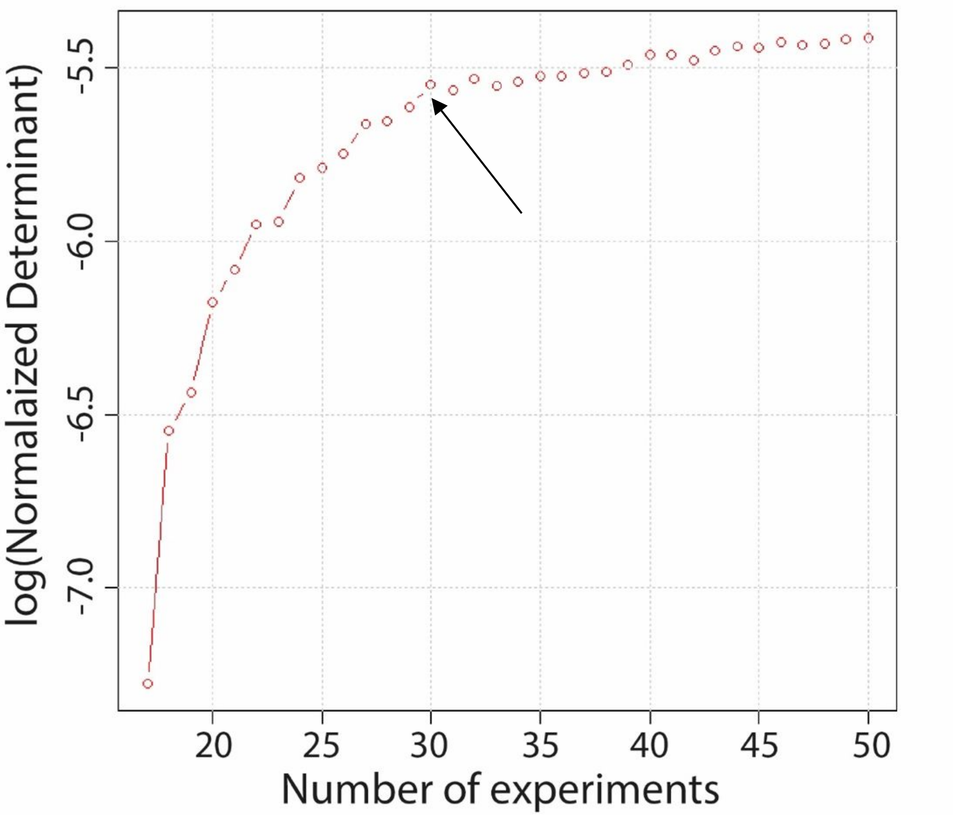

Figure S1: Plot of the log(Normalized Determinant) values versus the number of experiments.

All the performed experiments and the predicted response are shown in the Table 2S, all the experiments carried out in this study were performed in the presence of 50 nM of miR-29 and the results were expressed in terms of signal change %.

Table S2: Experimental plan and predicted responses as a consequence of the DO design.

Experiment	[Probe] (nM)	[NaCl] (mM)	Frequency (Hz)	Binding time (min)	Amplitude (V)	AuNPs (μL)	Predicted Y
1	100	50	10	60	0.01	2	23.35

2	100	50	100	10	0.04	2	21.44
3	1000	50	10	30	0.04	2	20.64
4	1000	50	100	60	0.04	2	9.95
5	1000	50	100	10	0.01	8	10.22
6	500	50	50	10	0.04	8	12.86
7	100	50	50	60	0.04	8	27.77
8	1000	50	10	10	0.01	15	9.69
9	100	50	100	10	0.01	15	22.53
10	100	50	10	30	0.01	15	31.32
11	1000	50	50	60	0.04	15	6.96
12	500	50	10	60	0.04	15	17.6
13	100	500	10	10	0.04	2	17.43
14	1000	500	50	10	0.04	2	6.61
15	1000	500	10	60	0.01	8	2.98
16	100	500	100	30	0.04	8	24.34
17	500	500	100	30	0.01	15	8.41
18	100	500	10	60	0.04	15	25.3
19	500	1000	10	10	0.01	2	7.54
20	100	1000	100	30	0.01	2	19.48
21	100	1000	50	60	0.01	2	13.45
22	1000	1000	50	60	0.01	2	2.82
23	1000	1000	100	10	0.01	8	10.48
24	500	1000	10	60	0.01	8	9.17
25	100	1000	10	10	0.04	8	24.54
26	100	1000	50	10	0.01	15	16.2
27	1000	1000	100	10	0.04	15	11.72
28	1000	1000	50	30	0.04	15	13.82
29	1000	1000	10	60	0.04	15	10.42
30	100	1000	100	60	0.04	15	24.28

Model validation

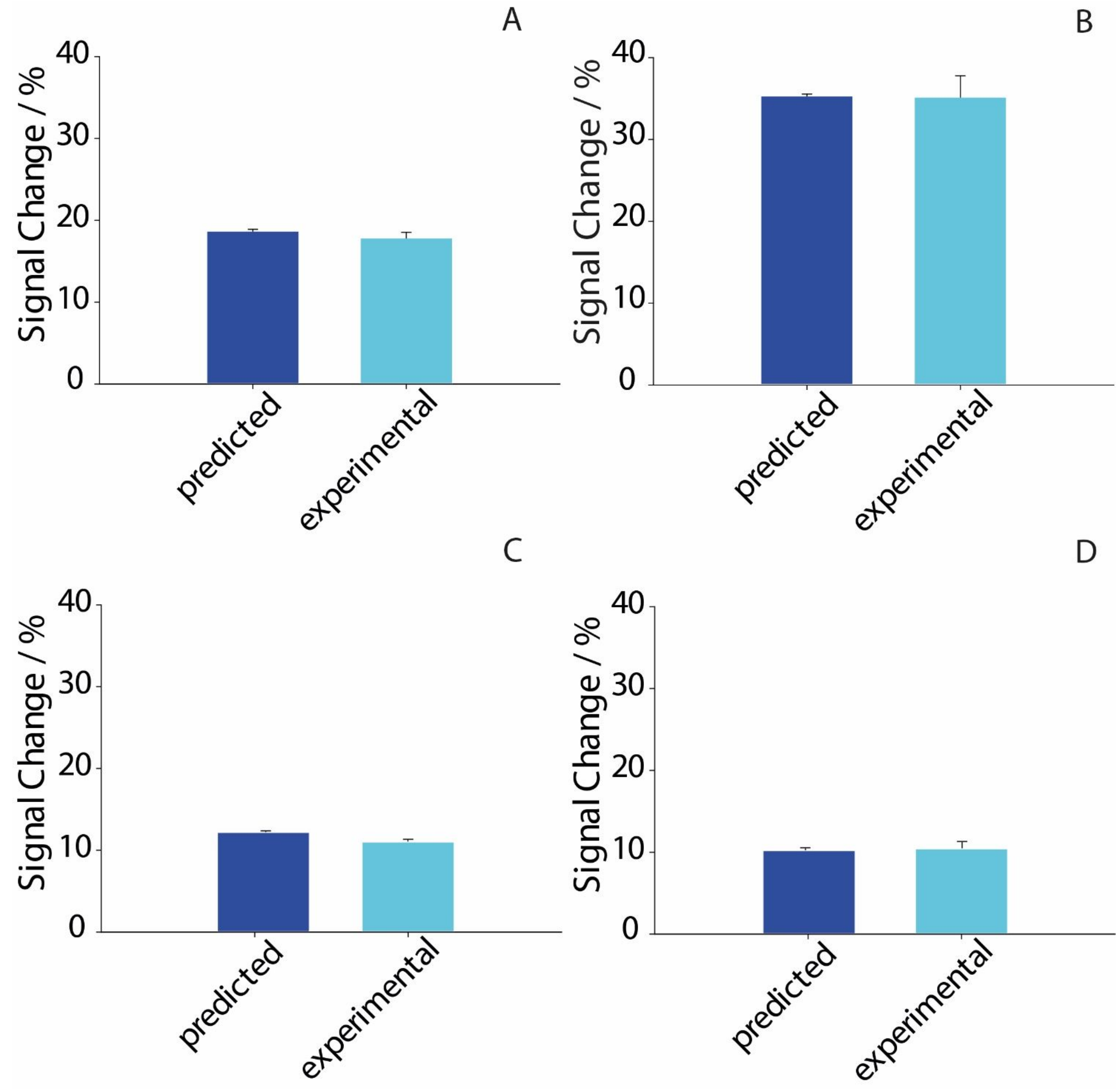

Figure S2: Comparison between predicted and experimental response: A) Protocol optimized with OVAT approach, with the parameters shown in table 3S. B) Protocol optimized with DoE approach, with the parameters shown in table 3S, C) predicted and experimental response obtained with the parameters shown in table 3S. D) predicted and experimental response obtained by the experiment shown in the table 3S. All the experiments were carried out in triplicate in the presence of 50 nM of target.

Table S3: Experimental plan for the model validation.

Experiment	[Probe] (nM)	[NaCl] (mM)	Frequency (Hz)	Binding time (min)	Amplitude (V)	AuNPs (µL)
A	100	140	50	30	0.01	8
B	100	50	10	30	0.036	11.5
C	500	50	10	10	0.01	8
D	1000	50	50	10	0.04	15
